# Different approaches for transformation of mesenchymal stem cells into hepatocyte-like cells

**DOI:** 10.1186/s13287-020-1555-8

**Published:** 2020-02-07

**Authors:** Afsoon Afshari, Sara Shamdani, Georges Uzan, Sina Naserian, Negar Azarpira

**Affiliations:** 1 Transplant Research Center, Shiraz University of Medical Sciences, Khalili street, Shiraz, Iran; 2 INSERM UMR-S-MD 1197/Ministry of the Armed Forces, Biomedical Research Institute of the Armed Forces (IRBA), Paul-Brousse Hospital Villejuif and CTSA Clamart, 94807 Villejuif, France; 3 SivanCell, Tehran, Iran; 4 CellMedEx, Saint Maur Des Fossés, France

**Keywords:** Mesenchymal stem cells, Hepatocyte-like cells, Transformation

## Abstract

Due to the prominent role of the liver in the body and detoxification, its functionality can be affected in an irreversible manner by diseases. This phenomenon renders the liver to stop working, leading to morbidity and mortality. Therefore, liver transplantation is the only way to tackle this issue.

In order to compensate for the lack of adequate healthy liver tissue for transplantation, therapeutic approaches such as hepatocyte transplantation have been proposed as an alternative. Recognizing the fact that mesenchymal stem cells are adult stem cells with the capacity to differentiate into several cell types, different methods have been invented to produce hepatocyte-like cells from mesenchymal stem cells. They can be divided into three main categories, such as addition of cytokines and growth factors, genetic modifications, and adjustment of microenvironment as well as physical parameters.

In this review, we attempted to introduce diverse efficient methods for differentiating mesenchymal stem cells and their capability for transformation into hepatocyte-like cells.

## Introduction

The liver is designed to accomplish many tissue-dependent tasks such as detoxification of drugs and endogenous substrates, as well as synthesis of plasma proteins and bile. Parenchymal hepatocytes, consisting of 80% total hepatic volume, are the major cells that carry out the aforementioned functions [1, 2]. For end-stage liver-related suffering patients, transplantation is the only possible way. Many of these patients in the waiting lists die before transplantation; hence, other therapeutic approaches like hepatocyte transplantation and bioartificial liver (BAL) devices can be proposed as alternative treatments [3].

In order to take advantage of fundamental researches in the clinic to treat various degenerative and autoimmune liver diseases, a reliable source of functional cells is warranted [4]. Having enough knowledge about liver formation and embryogenesis can contribute to the development of differentiation and reprogramming protocols. Hepatocytes can be differentiated from embryonic stem cells, induced pluripotent stem cells (iPSC), and mesenchymal stem cells (MSCs), providing a potential source of functional cells for transplantation.

Stem cells (SCs) are said to have three principal characteristics: the ability for self-renewal, differentiation into multiple cell lineages, and functional activity in a given tissue in vivo [5]*.* According to their origin, they are classified into two categories. The cells that are risen form blastocyst stage, which are called embryonic stem cells (ESCs), while the ones that compose niches of mature adult tissues and bone marrow are known as adult stem cells [5]. MSCs are stem cells that are well-known for their proliferation and differentiation abilities in vitro [6]. Friedenstein et al. were the first to isolate MSCs in 1968 from the bone marrow and introduced them to the scientific community [7].

There are a variety of sources that MSCs can be collected from, which makes them an outstanding supply in order to apply them for cell therapy in liver diseases. There are various approaches for differentiation of MSCs into hepatocyte-like cells (HLCs) [8–10]. Therefore, in this review, different assessment methods for differentiation of MSCs into HLCs are categorized, which might elucidate the best strategy for researches and further clinical scale-up in the future.

## MSC characteristics and sources

The International Society for Cellular Therapy (ISCT) has introduced criteria for the definition of MSCs including fibroblast-like morphology; plastic adherence; differentiation to adipocytes, osteoblasts, and chondroblasts; and positive expression of CD44, CD105, CD73, and CD90, with negative expression of CD45, CD34, and HLA-DR surface molecules [11]. It is worth mentioning that in vitro properties and surface molecular expressions might differ in MSCs from various origins [12].

**Fig. 1:**
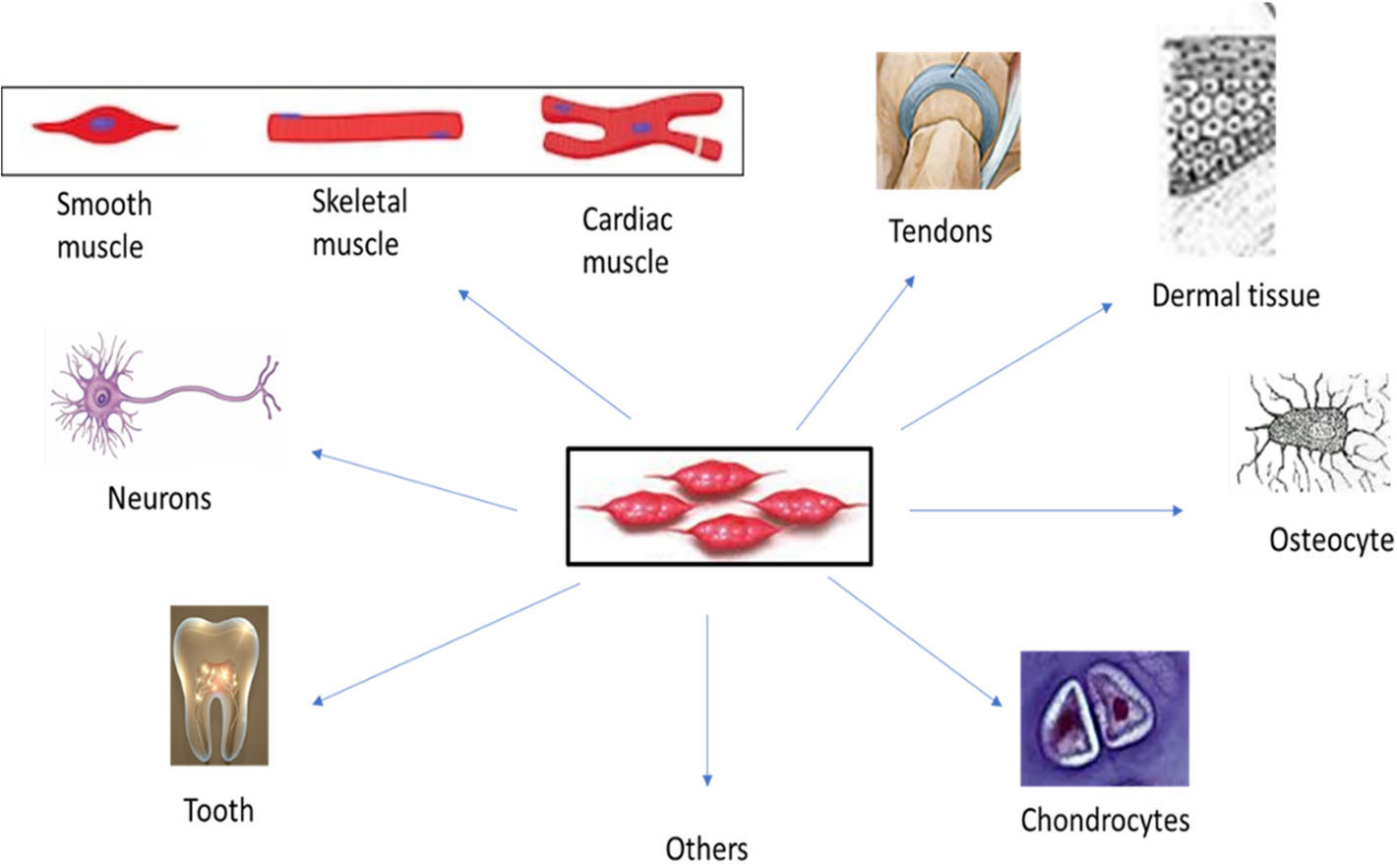
MSC differentiation capacities toward verity of cell lines

Due to some significant characteristics of MSCs, the research in this field is growing exponentially. One of these significant characteristics is somehow easy isolation methods that can be utilized for standard culturing of MSCs [10]. Another important criterion of MSCs is their immunomodulatory properties. They can produce many cytokines while not having immunogenic properties. MSCs do not express or merely express low quantities of MHC class I and II antigens. Additionally, they lack B7 family co-stimulatory molecules that are essential for initiating immune responses [12]. According to these features, MSCs can be considered as a universal stem cell source for transplantation without immunological rejections and need for immunosuppression drugs. Lastly, the other significant character of MSCs is their differentiation capacity. MSCs can be differentiated into other mesodermal cell types like chondrocytes, adipocytes, and osteocytes in response to specific stimuli. Even, they can be transdifferentiated into tissues of all three embryonic layers [13–15] (Fig. 1). This capacity proposes a great clinical potential in regenerative medicine. In Table 1 and Additional file 1: Table S1, a comparison of different MSC sources and their differentiation potentials is summarized. It was reported that MSCs derived from specific sources exhibit preference in their differentiation pattern and scientists are investigating how the origin of MSCs might affect their final differentiation program [26]. Therefore, the capacity of MSCs in tissue regeneration might be related to the tissue sources, which they were collected from [4].

**Table 1:** Summarizing studies that used growth factor and cytokines for differentiating MSCs into HLCs

MSC source	Cytokines and growth factors used for hepatic differentiation	Estimated properties of differentiated HLCs	References
AT-MSCs	HGF, FGF-1, FGF-4	Produce ALB, uptake low-density lipoprotein and ammonia detoxification, could incorporate in the parenchyma of the mouse liver after transplantation	[16]
AT-MSC	Using Dexa, ascorbic acid, EGF, bFGF, and HGF	Gene expression analysis, functional assays, and transplantation into mouse with chronic liver injury	[17]
AT-MSC	FGF, EGF, HGF, OSM, Dexa, and TSA	Hepatocyte-specific markers (ALB and AFP), bioactivity assays (LDL uptake and glycogen storage)	[18]
UC-MSCs	Sequential exposure to EGF, bFGF, bFGF-HGF, and finally OSM	Analyzed HLCs by reverse-transcription polymerase chain reaction, flow cytometry, and immunocytochemical assays	[19]
UC-MSCs	Sequential exposure to TSA or DMSO	Morphology and protein expression, urea synthesis, ammonia concentration	[20]
UC-MSCs	One-step protocol by using HGF and FGF-4	ALB, AFP, and CK-18, LDL uptake, and glycogen storage	[21]
UC-MSCs	Emphasizing on the critical role of OSM	Function of differentiated cell by PAS staining and LDL uptake was examined. The protein expressions of TP, ALB, GLB, BUN, and AFP were also detected	[22]
UCB-MSCs	HGF and FGF-4	Urea production and protein secretion and production of AFP and ALB	[2]
umbilical cord vein MSCs	Two-step protocol that contained HGF and OSM	Liver-specific protein markers such as ALB and CK-18 and expression of transthyretin, glucose 6-phosphatase, CK-18,18, AFP, hepatocyte nuclear factor-3β and ALB, indocyanine green cell uptake, glycogen storage	[23]
F-MSCs	HGF, bFGF, and OSM	Measured the expression of hepatocyte-specific markers such as AFP and CK-18	[24]
BM-MSCs	FGF-4, HGF, and combination of HGF-ITS-Dexa, and TSA	Glycogen storage and CK-18 expression, HNF-3beta, AFP, CK18, ALB, HNF1α, MRP2 and C/EBPα, ALB secretion, urea production and P450 (CYP)-dependent activity	[25]

Bone marrow MSCs (BM-MSCs) are typically derived from bone marrow biopsy of the iliac crest, and also other BM cavities [27], but this procedure is painful. It is important to mention that BM progenitor cells lose their proliferative capacity during the aging process, which contributes to a significant decrease in their differentiation potency after age 20 [14].

MSCs can be isolated from adipose tissue (AT-MSCs) from different parts of the body, and the most common source is the discarded fatty tissues after liposuction or plastic surgeries [28]. Dental pulp-MSCs (DP-MSCs) have the capacity to be differentiated more easily toward neuronal lineage and are used in surveys correlated to Parkinson’s disease as well as neurodegenerative diseases, spinal cord injuries, Alzheimer’s diseases, and stroke [14]. Heart-derived MSCs (H-MSCs) exhibit higher levels of cardiovascular-related markers such as myosin light chain-2a [29].

MSCs can be isolated from different birth-associated tissues such as the placenta, amnion, chorion, umbilical cord (UC), and cord blood (CB). Also, it is suggested that MSCs derived from neonatal sources have more differentiating capabilities in comparison to adult sources [30]. Some researchers have elucidated that human placenta-derived MSCs have higher expansion and engraftment capacity than BM-MSCs [31, 32]. Placenta- and amnion-derived MSCs express pluripotency-related genes like Oct-4 and Nanog. They can be duplicated over 250 times and have long telomers [13, 33]. Also, it is important to consider that OCT4 upstream region is important in pluripotency in early embryo development and the establishment of embryonic stem cells [34].

**Fig. 2:**
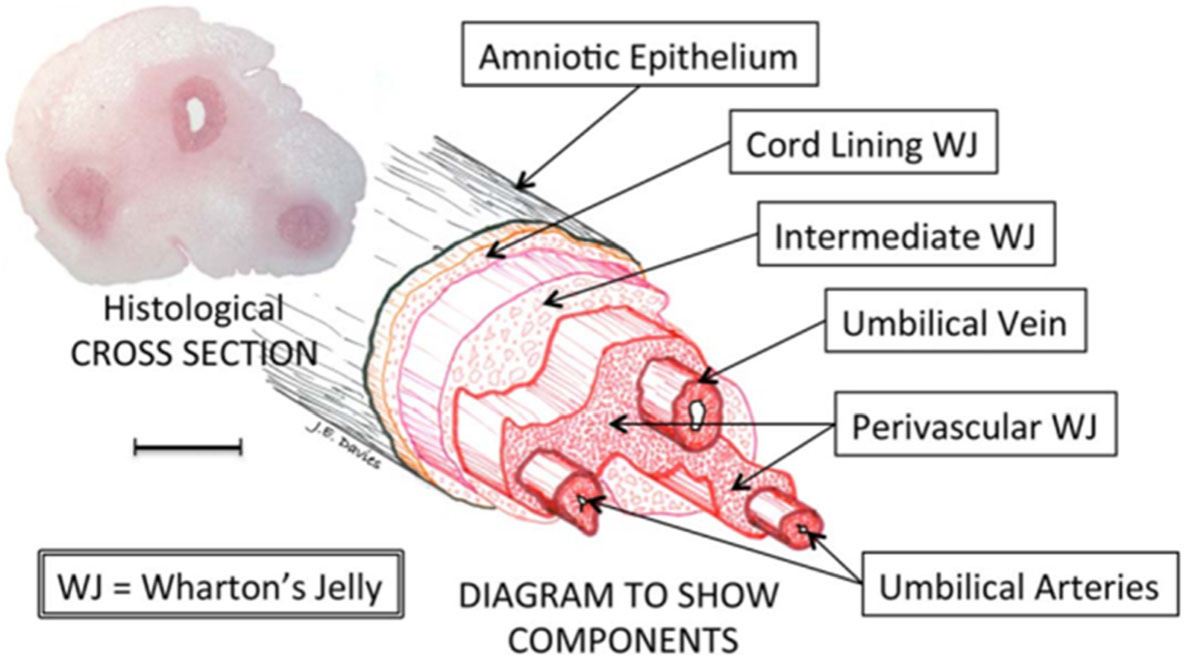
The structure of the human umbilical cord with a three-dimensional exploded diagram. The diagram is made by direct tracing the outlines of various features in the histological section, then shifting them along the tilted longitudinal axis. Scale bar = 5 mm [15]

Unlike peripheral blood that contains low number of MSCs if any, 10–30% of umbilical cord blood cells are composed of MSCs [14, 35]. These cells have high proliferative activity, and after several passages, they do not show any signs of senescence [36]. A jelly-like matrix material exists inside the cord that guards the cord arteries and the veins, which was initially described by Thomas Wharton in 1656 (“Wharton’s jelly”). Wharton’s jelly (WJ)-derived MSCs (Fig. 2) migrate through the developing cord, during embryogenesis, and some of these cells are trapped that can contribute to the cellular composition of the cord [13].

## The molecular embryogenesis of the liver

In the production of the liver parenchymal cells, the anterior part of the definitive endoderm (DE) is accountable, during gastrulation of the embryo. When the foregut closes, the progenitor cells which are trapped inside lie near the cells that create the heart and also regions of lateral plate mesoderm (LPM) that finally generate the proepicardium and septum transversum (STM). Fibroblast growth factor (FGF) and bone morphogenetic protein (BMP) are different temporally regulated signals from cardiac mesoderm and STM respectively, that are responsible for this induction [37]. Hepatic induction starts following an FGF signal, that activates the RAS/MAPK pathway. Several FGFs (FGF1, 2, 8, and 10) are detected in the cardiac mesoderm during the beginning steps of hepatogenesis [38].

**Fig. 3:**
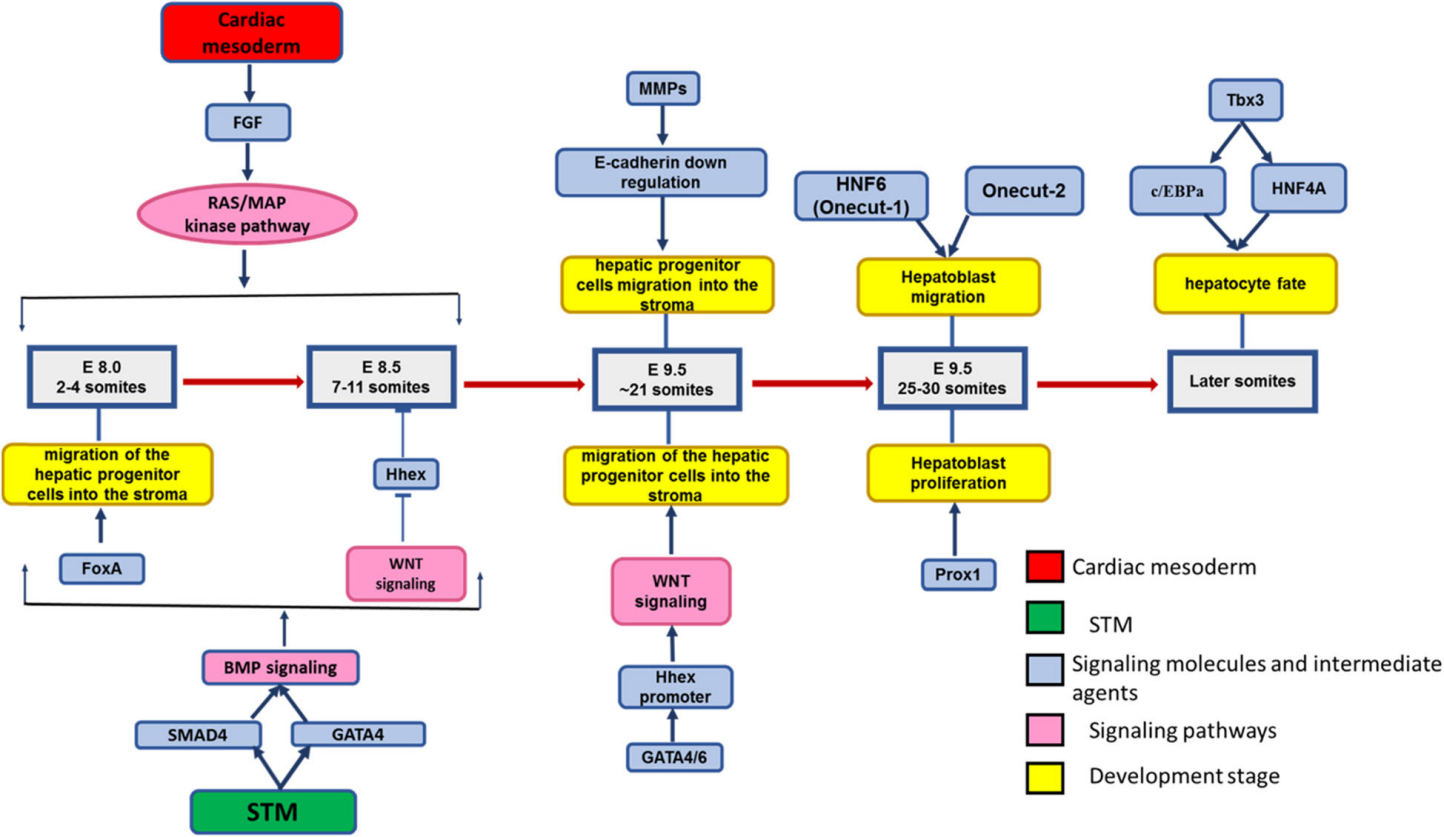
Molecular pathways in embryogenesis of the liver. Production of the liver parenchymal cells starts from the anterior part of the primary liver bud. FGF from cardiac mesoderm and BMP, which is mediated by SMAD4, interfere in hepatic induction through RAS/MAP kinase pathway and BMP signaling. GATA4 regulates expression of the secreted BMP4, which is highly expressed in the STM mesenchymal cells at the 8-somite stage. At early somite stages WNT signaling acts around 7–11 somites to repress the expression of Hhex. At around 21 somites, the matrix surrounding the basal surface of the epithelium is degraded, and E-cadherin expression is downregulated in the hepatic cells by the action of MMPs. GATA4 and/or GATA6 cause hepatoblast development by transactivating the Hhex promoter. Around 25 somites, Onecut-1 and Onecut-2 are redundantly essential for hepatoblast migration. Prox1 also promotes hepatoblast proliferation and migration from the primary liver bud. Tbx3 normally promotes a hepatocyte fate and represses a cholangiocyte fate through the expression of Hnf4a and c/EBPa. *FGF* fibroblast growth factor, *HNF* hepatocyte nuclear factor, *BMP* bone morphogenetic protein, *Fox A* Fork-head box protein A, *Hhex* hematopoietically expressed homeobox, *STM* septum transversum, *MMPs* matrix metalloproteinases, c*/EBPa* CCAAT-enhancer-binding proteins, *Tbx3* T-Box 3, Prox*1* prospero-related homeobox transcription factor

SMAD4 is the mediator of BMP signaling, which leads to histone, and subsequent transcriptional activation of liver-specific genes [39]. Studies in mouse embryos have shown that the hepatocyte nuclear factor beta (HNF1b) is one of the critical molecules during hepatic specification [40] (Fig. 3).

Liver bud is surround by STM mesenchymal cells that GATA4, a zinc finger transcription factor, is abundantly expressed inside them [41]. GATA4 regulates the expression of secreted BMP4 and is highly expressed like GATA4 in the STM mesenchymal cells at the 8-somite stage of mouse development [37]. Analyses in 2–4 somite stages revealed that Fork-head box protein A (FoxA) and GATA4 are expressed in the anterior endoderm and can attach to the albumin (ALB) enhancer before the onset of ALB expression [42], which following this attachment, the repositioning of nucleosomes happens [43].

In addition, the WNT signaling pathway gets involved during hepatic development through a very complex intervention. Studies have shown that canonical WNT signaling has different impacts, due to the developmental stage. In the primary stages of somite (4-6 somites) and in the posterior endoderm, expression of Hhex (Hematopoietically-expressed homeobox; a critical transcriptional regulator during hepatic development) is repressed as a result of WNT signaling action [44] (Fig. 3).

Hepatic differentiation is continued by active transcription of albumin regarding as the liver-specific gene in the primary stages of embryogenesis (day 8.5; E8.5) in the mouse ventral foregut endoderm, serving as the first line of evidence for hepatic differentiation [45]. And after that, the hepatic endoderm cells transform into a columnar morphology and start expressing some hepatic genes that are markers of early hepatic cell fate AFP (alpha fetoprotein), Ttr (transthyretin), Rbp (retinol binding protein), and Hnf4a, all of which are reliable indicators of early hepatic cell fate [46].

At around 21 somites in the mouse, following the onset of downregulation of E-cadherin expression, the degradation of the matrix covering the epithelium basal surface starts and these happenings result in the hepatic progenitor cells migration into the stroma (by the action of matrix metalloproteinases (MMPs)) occurs [47]. As previously mentioned, Hhex and WNT signaling participate in regulating the onset of hepatogenesis in different ways. At this stage, proliferation and positioning of the ventral endoderm which is placed in the cardiogenic field is regulated by Hhex. It seems that mentioned events regulate the induction of hepatic cell fate. In addition to mesodermal functions, which was already discussed, the transactivation of the Hhex promoter by GATA4 and/or GATA6 might contribute to hepatoblast development by transactivating the Hhex promoter [41].

Further transcriptional regulators have been considered due to their role in later events (around 25 somites and later). For instance, Onecut-1 (homeodomain factors HNF6) and Onecut-2 are redundantly essential for hepatoblast migration [47]. The prospero-related homeobox transcription factor (Prox1) also is responsible for hepatoblast proliferation and migration from the primary liver bud [48]. T-Box 3 (Tbx3) generally endorses a hepatocyte fate and suppresses cholangiocyte fate through the expression of Hnf4a and c/EBPa (CCAAT-enhancer-binding proteins) [49]. HNF4a controls apolipoprotein B and glucose metabolism through glucose-6-phosphatase and glycogen synthase, which both are essential for lipid metabolism [50].

During hepatogenesis, the hepatoblasts that migrate into the STM have the ability to differentiate into either cholangiocytes or hepatocytes. Cells that follow a hepatocyte cell fate progressively mature and make the liver bulk and the cholangiocytes make the biliary epithelial cells [51] (Fig. 3).

Around E10 of mouse development, migration of hematopoietic stem cells into the liver starts and hepatocyte maturation happens through signals from these hematopoietic cells, such as Oncostatin M (OSM) [52].

**Fig. 4:**
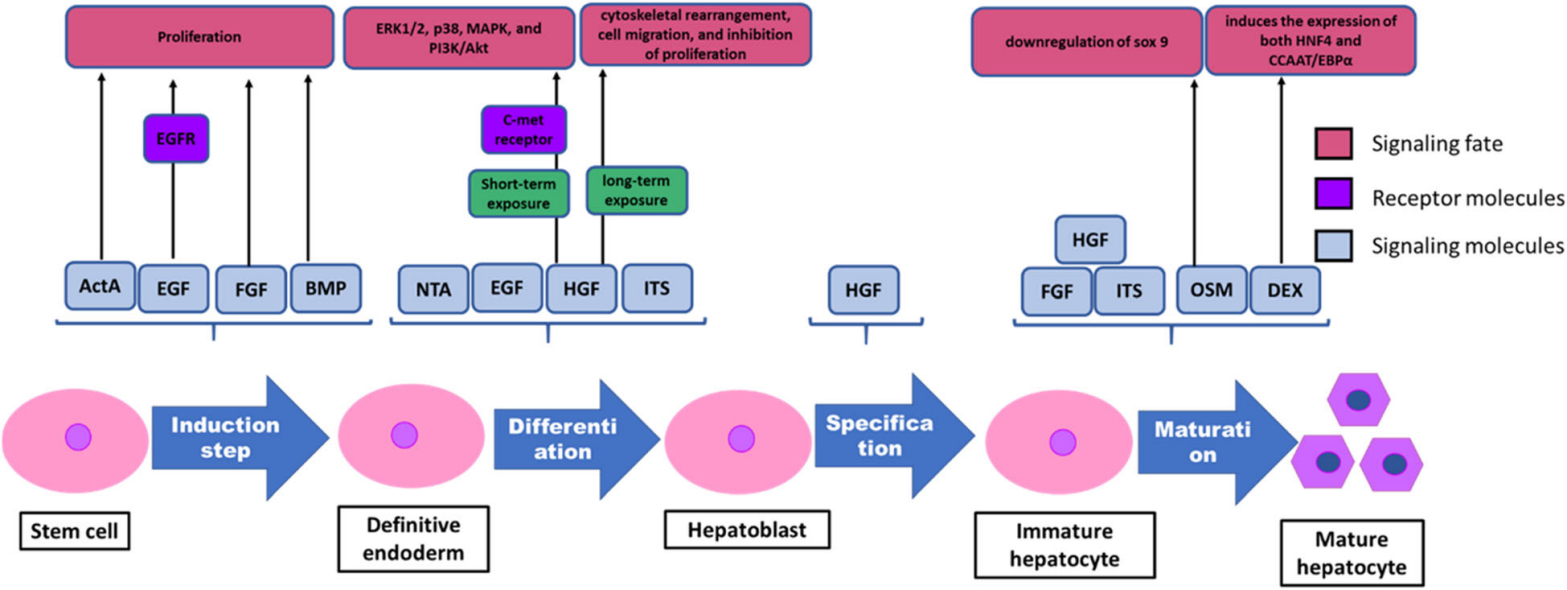
In vitro hepatic differentiation pattern. Hepatocytes can be differentiated from ES cells in vitro by mimicking the developmental processes of liver formation. Stepwise differentiation (ES cells → DE → hepatoblasts → immature hepatocytes → mature hepatocytes) adopted from liver developmental processes was applied to produce in vitro HLCs. *EGF* epidermal growth factor, *EGFR* epidermal growth factor receptor, *ERK1/2* extracellular-signal-regulated kinase 1/2, *FGF* fibroblast growth factor, *HGF* hepatocyte growth factor, *ITS* insulin-transferrin-selenium, *MAPK* mitogen-activated protein kinase, *OSM* oncostatin M, *PI3K* phosphoinositide 3-kinase, *NTA* nicotinamide, *ActA* Activin A, *BMP* bone morphogenetic protein

Therefore, according to different stages of hepatocyte maturation during embryogenesis, stepwise differentiation (ES cells → DE → hepatoblasts → immature hepatocytes → mature hepatocytes) adopted from liver developmental process was applied to produce in vitro HLCs (Fig. 4) (for review, visit ref. [16–25, 53–70]).

Using serum and other undefined culture medium components might be responsible for varying efficiency of hepatocyte differentiation from ES cells. Therefore, Si-Tayeb et al. introduced a hepatocyte differentiation protocol via reducing serum and application a well-defined culture medium components that can elicit the efficient and reproducible generation of hepatocytes from human ES [10] (for more examples ref. [16–25, 53–70]). They claim that using their protocol can help researchers to obtain more than 80% definitive endodermal cells, subsequently more than 80% hepatoblasts, and finally about 80% albumin-expressing hepatocytes (based on flow cytometry), indicating 80% efficiency of hepatocyte differentiation from human ES.

## Various in vitro techniques for the differentiation of MSCs into HLCs

According to studies, it is clarified that numerous signals through the extracellular matrix, growth factors, and even juxtacrine signals, which are produced by adjacent cells, regulate the cells’ behavior cooperatively [71]. The timing and distribution patterns of the mentioned signals are specific for each organ and developmental phase and are completely controlled. Consequently, it is vital to produce environments similar to the local environment for in vitro cultures to obtain more accurate results [72]. Previous reports revealed that MSCs derived from different sources can be differentiated into hepatocytes both in mice [73] and humans, using different protocols and techniques in vitro [9, 74].

### Addition of cytokines and growth factors

Knowing that the liver is derived from the DE and nodal, a member of the transforming growth factor β (TGFβ) superfamily signaling is essential for endoderm specification. During development, the ventral foregut endoderm receives BMP and FGF from its surrounding tissues and gives rise to hepatoblasts. Therefore, Activin A treatment is followed by BMP2/4 and FGF2/4 treatment [75]. For further maturation, hepatocyte growth factor (HGF), oncostatin M, dexamethasone, and/or EGF were added into the culture medium (reviewed in reference [76, 77]) (Fig. 4).

Understanding the fact that growth factors can selectively alter cell function and are capable of promoting MSC proliferation, migration, and differentiation formed the foundation for many researches in tissue engineering [53, 54]. Accordingly, investigators have attempted different protocols to differentiate these cells into other cell types such as hepatocytes. It is noteworthy to say that some researchers prefer to use sequential differentiation step by step (firstly induction and then maturation), using different growth factors, cytokines, and biochemicals that have roles in every stage-like development and regeneration [25, 55].

Hepatic differentiation protocol with Iscove’s modified Dulbecco’s medium (IMDM) and sequential cytokine supplements (Fig. 4) is the most popular hepatic differentiation procedure. In the primary induction step, the induction of MSCs into endodermal cells is carried out by EGF and FGF. EGF stimulates the proliferation of MSCs by binding to EGF receptor (EGFR) [56]. FGF belongs to a family that is composed of at least seven closely related polypeptides and, has a critical role during the onset of endodermal patterning. Amongst these, FGF-4 and basic FGF (bFGF) are used regularly. Similar to EGF, FGF also has impact on the proliferation rate of MSCs [57].

FGF, HGF, nicotinamide (NTA), and insulin-transferrin selenium (ITS) are normally added to cultures in order to induce cell differentiation. HGF is a mesenchymal origin pleiotropic cytokine, involved in the regulation of proliferation, differentiation, and chemotactic migration of MSCs [58]. Forte et al. [59] presented that when MSCs are exposed to HGF in a short-term manner c-met receptor and it’s downstream effectors such as ERK1/2, p38, MAPK, and PI3K/Akt are activated. However, the long-term exposure can cause cytoskeletal rearrangement, cell migration, and marked inhibition of proliferation. Furthermore, ITS and NTA promote the proliferation and survival of primary hepatocytes [60].

OSM and dexamethasone (Dexa) are required to induce further maturation, together with the addition of FGF, ITS, and HGF. OSM plays an important role in progression of liver maturation from developing hepatocyte [61]. A study specified that the hepatic induction effects of OSM might be related to downregulation of sox, which enforces the proliferation by maintaining the pluripotency of stem/progenitor cells [62]. Dexa can induce the expression of both HNF4 and CCAAT/EBPα. These two mentioned transcription factors are crucial for differentiation of hepatocytes [63]. Also, trichostatin A and sodium butyrate that are histone deacetylase inhibitors contribute to stem cell hepatic differentiation. As a matter of fact, the expression of hepatocyte-specific genes and functions might be enhanced through histone deacetylase inhibitors [25, 64].

In this regard, there are multistep procedures that treat cells by growth factors in different cell sources. Some protocols prefer to separately add growth factor and cytokines. For instance, they used BMP and FGF family, followed by utilization of Dexa and OSM (sequentially) [16, 63]. Others have used single-step procedures that simultaneously expose cells to growth factors like HGF and EGF [65, 66]. Also, there are protocols that use a combination of both proliferation (such as EGF and HGF) and maturation factors (such as OSM, Dexa, and ITS) in two separate steps [9]. Table 1 summarizes some of these studies.

Some researchers chose AT-MSCs as the HLC differentiation source. They deduct that this source is multipotent fraction of adherent cells that attach to culture wares and remain there as a heterogenous population of fibroblast-like cells. They are similar to BM-MSCs in differentiation potential and, while being heterogenous, reveal a surface antigen marker profile [17–20, 66].

Banas et al. collected AT-MSCs from different age group patients and used growth factors like HGF, FGF1, and FGF-4 to differentiate them into hepatocytes. Their results showed that ACD105+ AT-MSCs has high hepatic differentiation ability and the differentiated HLCs could produce ALB, with the ability to uptake low-density lipoprotein (LDL) and ammonia detoxification. Also, these cells could be incorporated in the parenchyma of the liver after transplantation into mice [16]. In another research, a method was established to generate functional HLC clusters, using the floating culture method, which induces functional HLC clusters that function efficiently both in-vitro and in-vivo. The characteristics of produced HLC clusters were analyzed by gene expression analysis, functional assays, and transplantation into mouse afflicted with chronic liver injury. The result showed that cell clusters can express various genes that are commonly found on mature hepatocytes, and also displayed functional characteristics of hepatocytes such as ALB expression, urea secretion, cytochrome P450 activity, take up LDL, and stored glycogen. Also, they stated that after transplanting of these cell clusters into chronic liver injured mouse the serum levels of ALB and total bilirubin improved significantly [17]. The objective of Yin et al. was to observe the hepatic differentiation ability of AT-MSCs in vivo and in vitro. The differentiated cells showed HLC morphology and hepatocyte-specific markers (ALB and AFP) and bioactivity assays (LDL uptake and glycogen storage). ALB was detected in the mouse livers 1-month post-transplantation. Furthermore, their study revealed that trichostatin A (TSA) enhanced ALB production and LDL uptake by the HLCs, which was compared with the functions of human liver cells [18].

Another source of MSCs is UC-MSCs, capable of differentiating into other cell types. Moreover, in vivo UC-MSC transplantation experiments introduced these cells as a promising candidate for restoration functional defects in some injuries and diseases [21–24].

For the first time, Campard et al. isolated UC-MSCs and differentiated them into HLCs, by sequential exposure to EGF, bFGF, bFGF-HGF, and finally OSM. They analyzed HLCs by reverse-transcription polymerase chain reaction, flow cytometry, and immunocytochemical assays, and then compared with undifferentiated UC-MSCs as well as freshly isolated liver cells. They concluded that UC-MSCs, with an endodermic differentiation capacity, might be an alternative source for liver-directed cell therapies. They showed that in vitro undifferentiated UC-MSCs constitutively expressed hepatic markers, including ALB, AFP, ck-19, connexin-32 (Cnx-32), and dipeptidyl peptidase IV (DPPIV). Also, the newly differentiated cells showed hepatocyte-like morphology, up-regulated several hepatic markers, stored glycogen, produced urea, and exhibited an inducible CYP 3A4 activity. Still, some hepatic markers were not found such as HepPar1 or HNF-4, which this means that their differentiation level was not reached to that of mature hepatocytes. They also, observed that some of the MSC markers were partially preserved in the differentiated UCMSCs. Finally, the differentiated UCMSCs had the capacity of engrafting, and expressing of human ALB and AFP after 2, 4, and 6 weeks post transplantation in mouse livers, while cytokeratin 19 was completely downregulated [19]. Also, Yoon et al. examined the four-step sequential exposure of UC-MSCs with OSM plus TSA or OSM, plus dimethyl sulfoxide (DMSO) for in vitro differentiation into HLCs. Consequently, the morphology and protein expression had successively changed in a step-dependent manner. The urea synthesis and ammonia concentration rates of (OSM + TSA)- and (OSM + DMSO)-treated cells were measured [20]. In another study, UC-MSCs were differentiated into HLCs in a one-step protocol, using HGF and FGF-4. The analysis showed that these cells produced hepatocyte-specific markers ALB, AFP, and CK-18; stored glycogen; and uptook LDL [21]. Zheng et al. conducted a research, which showed the in vitro ability of UC-MSCs to differentiate into HLCs by emphasizing on the critical role of OSM by a modified differentiation protocol. The function of differentiated cells was examined via Periodic acid-Schiff (PAS) staining and LDL uptake. Also, the protein expressions of total protein (TP), ALB, globulin (GLB), urea (BUN), and AFP were also measured [22].

Hasan et al. differentiated UCB-MSCs into functional HLCs under hepatogenic conditions (containing HGF and FGF-4). The function of differentiated HLCs was detected by estimating urea production and protein secretion. The cells started production of AFP from day 7, and ALB gene was measured on the 14th day. This study exhibited that UCB-derived MSCs had the ability to differentiate into functioning HLCs starting on the 7th day after culturing under hepatogenic conditions, which became well-functioning on the 21st and 28th day [2].

In a study by Raufi et al., hepatic differentiation of umbilical cord vein MSCs was performed, using HGF and OSM in a two-step protocol by a 4-week induction. Immunological analysis indicated that derived HLCs expressed liver-specific protein markers such as ALB and CK-18 and also exhibited several characteristics of hepatocytes, including expression of transthyretin, glucose 6-phosphatase, CK-18, AFP, HNF-3β, and ALB. Also, the ability of indocyanine green cell uptake and glycogen storage was surveyed [23]. Furthermore, there are more studies that have used UCB-MSCs to differentiate into HLCs, using various combinations of cytokines and growth factors such as FGF, HGF, OSM, EGF, LIF, and ITS premix [67, 68].

Wei et al. used human fetal mesenchymal stem cells (F-MSCs) which were derived from the fetal bone marrow in order to differentiate them into HLCs. For this reason, they used HGF, bFGF, and OSM, and after 21 days of differentiation, the expression of hepatocyte-specific markers such as AFP and CK-18 by immunofluorescence staining were measured [24].

A study by Snykers et al. described that BM-MSCs cannot be differentiated into HLCs without TSA after sequential exposure to growth factors, especially in the final step, and they supposed that adding FGF-4, HGF, and combination of HGF-ITS-Dexa sequentially can reflect the extracellular signaling pattern during hepatogenesis. The differentiated cells induced both glycogen storage and CK-18 expression.

When the cells are additionally exposed to TSA, endodermal differentiation was improved by changes in epithelial morphology; chronological expression of hepatic proteins, including HNF-3β, AFP, CK-18, ALB, HNF1α, multidrug resistance-associated protein (MRP)2 and C/EBPα; and functional maturation, such as ALB secretion, urea production, and cytochrome P450 (CYP)-dependent activity [25].

All in all, it seems that different sources of MSCs can be used for transformation into HLCs. The need for a comprehensive study that compares the capacity of all the sources in differentiating into HLCs with each other is warranted. However, the existing data cannot elucidate as to which source is more suitable for developing functional HLCs to satisfy the need of a promising source for transplantation.

### Genetic modifications

#### MicroRNAs (miRs)

miRs are highly conserved, non-coding RNA molecules that are about 20 nucleotides in lengths that can repress or stimulate the translation or degradation of its target mRNAs. miRNAs can act as key regulators to determine cell fate. A combination of different miRs is responsible for the regulation of MSC differentiation [69].

Davoodian et al. studied the elucidating role of miR-122 during hepatic differentiation and showed that this process could be improved by the overexpression of miR-122. Their study confirmed that this miR can act as a prominent factor in differentiation of human AT-MSCs into HLCs [70].

Cui et al. studied the dynamic microRNA profiles of human UC-MSCs into hepatocytes. Hepatocyte formation was induced in-vitro After isolating hUC- MSCs, using growth factors. They induced hUC-MSCs for 26 days and differentiated cells could express hepatocyte-specific genes, synthesize glycogen and urea, and uptake LDL. Cellular total RNA from hUC-MSCs and hepatic differentiated hUC-MSCs was collected at seven time points, including days 2, 6, 10, 14, 22, and 26, for microRNA microarray analysis. The dynamic microRNA profiles that were detected did not have any overlap or only had partial overlap with microRNAs, which were reported to be involved in hepatocyte regeneration or hepatic differentiation of liver-derived progenitor cells. Sixty-one microRNAs out of 1205 human microRNAs exhibited steady changes and were altered at least by 2-fold between hUC-MSCs and differentiated cells microRNAs, and 25 of them showed over-expression. Also, 36 microRNAs were under-expressed and had a similar expression pattern to that of the overexpressed microRNAs. They elucidated that the microRNAs related to hepatic differentiation were not enriched in hepatocyte or hepatocellular carcinoma cells and might possibly have targets among liver-enriched transcription factors and genes [78]. In continuation, they clarified that six overexpressed miRNAs (miR-1246, miR-1290, miR-148a, miR-30a, miR-424 and miR-542-5p) have instructive role during hepatic differentiation of hUC-MSCs. Before hepatic differentiation, lentivirus holding a miRNA inhibitor sequence were used for infecting derived human MSCs. They found that downregulating of any one of the six-hepatic differentiation-specific miRNAs that mentioned could prevent HGF-induced hepatic differentiation including albumin expression and LDL uptake. While, it was detected that overexpression of any one of the six miRNAs alone or liver-enriched miR-122 cannot onset hepatic differentiation. Then, it was observed that ectopic overexpression of seven miRNAs (miR-1246, miR-1290, miR-148a, miR-30a, miR-424, miR-542-5p together with miR-122) simultaneously can stimulate human MSC conversion into functionally mature induced hepatocytes (iHep) [79]. Moreover, the induced iHep cells were transplanted into CCL4 liver injured mice and it was observed that iHeps not only could improve liver function but also restored the injured livers. Finally, they showed that the differentiated cells can improve liver injury in an animal model. In their studies, hMSCs were directly converted into hepatocytes using a seven-miRNA combination and they claimed that in comparison with conventional methods, the seven-miRNA combination promoted hepatic differentiation of hMSCs more quickly and efficiently [79, 80]. In another study, Khosravi et al. used miR-106a, miR-574-3p, and miR-451 for hepatic differentiation for in vitro UC-MSCs and confirmed the production of ALB [81]. The findings from these studies specify that miRNAs have the ability to directly convert human MSCs to a hepatocyte phenotype in vitro.

### Adjustment of microenvironment and physical parameters

In vivo, cells create communities in a three-dimensional (3D) microenvironment, where factors like interaction with neighboring cells, the extracellular matrix (ECM), and systemic factors are involved in determining their phenotype [82]. In addition, there are some common qualities within most mammalian cell microenvironments, such as continuous nutrient supply, short distances between cells, waste removal, and a definite temperature.

#### 3D scaffolds

The in-vivo environment cannot be represented correctly by traditional culturing methods on plastic culture wares; hence a paradigm shift from 2D to 3D experimental techniques is essential for fulfilling this need, different equivalents of natural, synthetic and semisynthetic ECM have been established to produce a suitable cellular microenvironment [83]. Standard 2D culture conditions which soluble growth factors are at uncommon high concentrations, make poor mimics of the cellular environment, because 3D signals are generally absent, oxygen tension is too high and adjacent cell interactions are not organized properly [84]. Most studies conducted on hepatic trans-differentiation of adult stem cells were accomplished in monolayer 2D culture, but it is identified that hepatocytes sustain their differentiated functions better in 3D multicellular aggregates or spheroids than in monolayer culture [85].

The aim of Ong et al. study was to study the hepatic differentiation capacity of MSCs in two types of 3D constructs: a cell pellet and a cell pellet accompanied by ECM from the small intestinal submucosa (SIS). SIS is a biomaterial derived from the porcine small intestine that contains collagen I, small quantities of collagen IV, fibronectin, and laminin. It seems that because SIS is composed of ECM molecules in its native state and concentration, it might have the potential to produce an appropriate biological signal to guide hepatic differentiation in-vitro. Moreover, SIS could provide angiogenesis in- vivo, which is vital for the survival of transplanted cells. In their study, the MSCs were cultured in two different constructs with growth factors, which confirmed successful hepatic differentiation. Finally, due to possible confounding factors of diffusion limitation, they showed that the SIS biomaterial is not advantageous enough for hepatic differentiation [86]. Pulavendran et al. incorporated HGF into biocompatible chitosan nanoparticles (CNP) and hepatic differentiation of MSC in the presence of HGF and CNP was explored in vitro [87].

In another study, Wang et al. produced a perfusion system for 3D cell culture and the porous polylactic glycolic acid (PLGA) polymer was used as the biodegradable scaffold. Human MSCs were seeded and proliferated in PLGA scaffolds, and a medium containing HGF, FGF, and Dex was used to induce hepatogenesis. The comparison between perfused and statically induced differentiated human MSC survival and hepatogenic ability showed that perfusion is the reason of increased survival and the uniform distribution pattern of induced cells in the scaffolds, which resulted in a higher efficiency of hepatogenesis in the PLGA construct with hMSCs. Finally, they concluded that the oscillatory perfusion induction system combined with the hepatogenic medium can be a valuable and convenient tool for in vitro hepatic tissue engineering using human MSCs [88].

Khodabandeh et al. study aimed to evaluate the ability of human UC-MSCs as an accessible and achievable source for differentiation toward HLCs by a 3D culture system in the presence of hepatogenic media containing IGF-1 with or without FGF4. Following the pre-exposer of the cells into FGF4 before being treated with IGF-1 and HGF in 3D collagen scaffold, liver-specific marker expression had increased. Long-term culturing of UC-MSCs in 3D environment induced the cells to express higher level of hepatogenic markers at protein level, and also the induced cells became functional, as well [89].

Another study investigated the potential of UB-MSCs to turn into HLCs based on a sequential 2D and scaffold-based 3D differentiation protocol. For hepatic differentiation, a sequential 4-step protocol in 3D and 2D culture systems were used for induction of the cells. Urea concentration and ALB secretion were measured, and also, gene expression levels of AFP, ALB, and CK18 were determined. Morphological changes from elongated fibroblast-like cells to round epithelial cells were observed in 2D culture, using growth factors. Finally, their results showed that sequential exposures of UC-MSCs to growth factors in the 3D culture can improve hepatic differentiation [90].

#### Microfluidic devices

Microfluidic devices have been utilized for cell culture, cell differentiation [91], dynamic gene expression [92], and test of cellular response to chemical gradients [93]. According to reports, similar induction protocols for hepatic differentiation of MSCs have been used in many researches [94, 95]. However, these protocols require at least 4–6 weeks to complete the hepatic differentiation process; in addition, in these studies, large numbers of hepatocytes, which are necessary for cell therapy, are not fully addressed [96]. Therefore, it seems valuable to investigate hepatic differentiation of MSCs under continuous fluid flow, using a microfluidic device which mimics the shear flow in the microenvironment. Fluid shear stress, like blood pressure effect, is important in liver tissue development [97]. Therefore, microfluidic systems are used to simulate the condition of in vitro systems much more like the in vivo systems, using the diffusive mixing possibilities of the laminar flow and concentration gradients [84].

Therefore, to create a more in vivo-like 3D systems, further innovative approaches are essential that can be achieved through micro technology. Using microfluidic cell culture systems enables researchers to control the surrounding environment of individual cells, which is not possible via traditional culturing conditions (Table 2) [98].

**Table 2:** Properties and benefits of cell culture in microfluidic devices [97]

Property of microfluidic systems	Benefit for cell culture
Small chip size and microchannels on the cellular length scale (5–500 μm)	-Reduced sample/reagent consumption -Numerous cell coculture in a single device -Faster transfer of cell culture medium and heat, i.e., short equilibration time
2D or 3D network and structure	-Simulating in vivo cell growth development
The feasibility to integrate multiple microfluidic devices on a chip	-Integrate with fluid handling operations for efficient and high-throughput cellular analysis -Integrate with detection functionality for in situ monitoring of cellular events -Integrate with functionality for temperature control of the cellular microenvironment etc.

There are shortcomings when using conventional microfluidic devices for hepatic differentiation such as an uneven flow in a small culture chamber, which might affect the yield and quality of differentiated cells [91]. An uneven cell distribution which requires cell injection through seeding microchannels can affect cellular interaction [99]. To remove these problems, Yen et al. invented a novel biomicrofluidic device that could minimize the mentioned problems. They construct a five-layered microfluidic device which was composed of one layer of PS, patterned PDMS, patterned glass, and PMMA adaptors was designed and fabricated. The constructed device in cooperation with the other components of the biomicrofluidic system, applied for the maintenance and hepatic differentiation of MSCs effectively. They tried to maintain a constant flow rate and homogeneous medium flow of media in the cell culture chamber. They simulated field of velocity distribution in order to present a uniform flow profile in the culture region of the microfluidic device. In addition, they demonstrated that the flow field near the center line and the margin was the same. The Reynolds number of the microfluidic device for a 100-μl/h flow rate was 3.6 × 10^−5^. The flow is considered laminar flow when Re is smaller than 2100 and turbulent flow when larger than 4000. The 100-μl/h flow rate in our device was therefore a laminar flow. By using a two-step protocol and adding cytokines and growth factors, MSCs were differentiated to HLCs on a fabricated microfluidic device. The device consisted of a large culture chamber with an ability to create a stable and uniform flow to produce homogeneous induction of hepatic differentiation. Since the device was able to make HLCs efficiently, they are convinced that their structure can be used for scale-up production of HLCs from MSCs for cellular therapy [96].

#### Bioreactors

The liver continuously suffers from being exposed to a wide range of exogenous substances. Therefore, it is vital to produce cellular models that can mimic hepatic function that resembles in vivo conditions [100]. The progress made in the field of tissue engineering and micro technology has improved in the form of new tools entitled “microfluidic bioreactors,” which can be applied in many different fields [101].

In their study, Rebelo et al. manufactured a stirred-tank bioreactor with perfusion operation mode. They cultivated human BM-MSCs with hepatocytes isolated from human liver tissue as spheroids in an automated, computer-controlled bioreactor. Their study resulted in an inner core of parenchymal liver tissue with an outer layer of stromal cells. Hepatocyte polarization and morphology as well as the mesenchymal phenotype of BM-MSCs were maintained throughout the culture period, and the crosstalk between the two cell types was depicted. The viability, compact morphology, and phenotypic stability of hepatocytes were enhanced in co-cultures in comparison to monocultures. Gene expression and CYP3A4 and CYP1A2 activity was inducible until week 2 of culturing [102].

In another study, the first stage of hepatic differentiation of hn-MSCs (human neonatal mesenchymal stem cells) was performed in 2D monolayer cultures for 17 days. Then, cells were cultured in 3D as self-assembled spheroids or in multicompartment membrane bioreactor system as the second stage. The system enabled hn-MSC differentiation into HLCs which was certified by positive immune staining of hepatic markers, the hepatic transporters, and drug-metabolizing enzymes. In addition, all models displayed relevant glucose metabolism, ALB production, and urea secretion. These data suggest that the tested 3D models could improve HLC maturation, showing a relevant biotransformation capacity, by providing more reliable models for mechanistic studies and more predictive systems for in vitro toxicology applications [103].

## Conclusion

Mesenchymal stem cells have the capacity to multiply and differentiate into numerous types of cell lineages including HLCs. Access to an abundant, high-quality supply of hepatocytes with therapeutic potential for cell transplantation and extracorporeal support of patients in liver failure is an important issue. Although many preclinical and clinical trials were performed to elicit their role in the hepatic regeneration, the best and most effective protocol for hepatocyte differentiation is still unknown. In this review, we suggested that MSC from different origins might be considered as a suitable source for differentiation into HLCs by different protocols. It seems that in the early stages of differentiation, 2D culture is sufficient, but during the development stage, 3D culture system with HGF and FGF cytokines will be more effective.

Although the number of HLCs is critical for transplantation, the functionality of these cells has an important clinical point of view. Different protein secretion (like urea production, ALB, and AFP) and enzymatic functions (like LDL uptake, glycogen storage, and cytochrome P450) can be tested for evaluation of HLCs in order to estimate their ability in renewing the damaged liver tissue. By the way, after using the HLCs in clinical trials, the routine method tests the liver function as well as considers the MELD score. The MELD score is composed of bilirubin level, which shows how well the liver clears the bile; INR (international normalized ratio), which reflects how well the liver makes coagulative factors needed for blood clots; and also serum sodium level.

Nonetheless, it is worth mentioning that MSCs might provide a reliable source of HLCs by reducing the gap between the availability and the demand for liver donors in the future.

## Supplementary information

Additional file 1: **Table S1.** Comparison of different MSCs sources in differentiating potentials.

## Data Availability

Not applicable

## Abbreviations

BAL: Bioartificial liver
iPSC: Induced pluripotent stem cells
MSCs: Mesenchymal stem cells
SCs: Stem cells
ESCs: Embryonic stem cells
HLCs: Hepatocyte-like cells
ISCT: The International Society for Cellular Therapy
BM-MSCs: Bone marrow MSCs
AT-MSCs: Adipose tissue MSCs
DP-MSCs: Dental pulp-MSCs
H-MSCs: Heart-derived MSCs
UC: Umbilical cord
CB: Cord blood
WJ: Wharton’s jelly
DE: Definitive endoderm
LPM: Lateral plate mesoderm
STM: Septum transversum
FGF: Fibroblast growth factor
BMP: Bone morphogenetic protein
TGFβ: Transforming growth factor β
HNF1b: Hepatocyte nuclear factor beta
FoxA: Fork-head box protein A
ALB: Albumin
AFP: Alpha fetoprotein
Ttr: Transthyretin
Rbp: Retinol binding protein
MMPs: Matrix metalloproteinases
Prox1: Prospero-related homeobox transcription factor
Tbx3: T-Box 3
c/EBPa: CCAAT-enhancer-binding proteins
OSM: Oncostatin M
LIF: Leukemia inhibitory factor
HGF: Hepatocyte growth factor
IMDM: Iscove’s modified Dulbecco’s medium
EGFR: EGF receptor
bFGF: Basic FGF
NTA: Nicotinamide
ITS: Insulin-transferrin selenium
Hhex: Hematopoietically expressed homeobox
Dexa: Dexamethasone
LDL: Low-density lipoprotein
TSA: Trichostatin A
Cnx-32: Connexin-32
DPPIV: Dipeptidyl peptidase IV
DMSO: Dimethyl sulfoxide
PAS: Periodic acid-Schiff
TP: Total protein
GLB: Globulin
F-MSCs: Fetal mesenchymal stem cells
MRP: Multidrug resistance-associated protein
CYP: Cytochrome P450
miRs: MicroRNAs
iHep: Induced hepatocytes
3D: Three-dimensional
ECM: Extracellular matrix
SIS: Small intestinal submucosa
CNP: Chitosan nanoparticles
hn-MSCs: Human neonatal mesenchymal stem cells
