# Supplementary material for: Different approaches for transformation of mesenchymal stem cells into hepatocyte-like cells
Source: Stem Cell Res Ther. 2020 Feb 7;11:54. doi: 10.1186/s13287-020-1555-8 (PMC7007672; doi:10.1186/s13287-020-1555-8)
Supplement: Supplementary file 1 — Table S1. Comparison of different MSCs sources in differentiating potentials. [file 13287_2020_1555_MOESM1_ESM.docx]

Table S1- Comparison of different MSCs sources in differentiating potentials

| MSCs source | Specific differentiating properties | Compare to | Ref. |
| --- | --- | --- | --- |
| BM -MSCs | Higher osteogenic and chondrogenic differentiation potential | AT-MSCs | (1) |
| BM-MSCs | Synthesis of collagen II and proteoglycan in growth factor treated BM-MSCs | AT-MSCs | (2) |
| BM-MSCs | More adipogenic and osteogenic and less chondrogenic differentiation potential | Peripheral blood-MSCs | (3) |
| BM-MSCs and AT-MSCs | More adipogenic differentiation potential and less lipid vacuoles production | CB-MSCs and PL-MSCs | (4,5) |
| CB-MSCs | Lack adipogenic differentiation potential | - | (6) |
| CB-MSCs | Differentiate into fibrocartilage with distinctly different morphology | BM-MSCs and PL-MSCs | (6) |
| BM-MSCs | Higher chondrogenic differentiation potential | PL-MSCs and lung MSCs | (7) |
| Fetal BM-MSCs | More calcium production and higher osteogenic- specific gene expression | Adult BM-MSCs | (8) |
| Fetal BM-MSCs | Higher osteogenic differentiation potential | Fetal blood-MSCs>fetal liver MSCs | (8) |
| AT-MSCs | Comparable potential to differentiate toward functional hepatocyte expressing key metabolic enzymes like P450s | BM-MSCs | (9) |
| AF-MSCs | Greater differentiation potential toward hepatocytes | BM-MSCs | (10) |
| PL-MSCs | Produce more hepatogenic cytokines like HGF | BM-MSCs and AT-MSCs | (11) |
| BM-MSCs | Both can differentiate to ILCs but I-MSCs produce more glucose-induced insulin | I-MSCs | (12) |
| BM-MSCs | Higher capacity to differentiate to ILC | AT-MSCs | (13) |
| BM-MSCs | Smaller ILC clusters and less insulin production | Primitive stromal cells derived from UC | (14) |
| NSs derived from AF-MSCs | Larger and higher proliferated neural cells/produce more neuronal stemness markers/produce more neurons and glial cells | NSs derived from BM-MSCs | (15) |
| WJ-MSCs | Better efficiency in neuronal induction | BM-MSCs and AT-MSCs | (16) |
| WJ-MSCs | Comparable potential to differentiate to dopaminergic neurons | BM-MSCs | (17) |
| NSs derived from AT-MSCs | greater expansion and diﬀerentiation abilities | NSs derived from BM-MSCs | (18) |
| UC-MSCs | significantly higher tubule length, diameter and area after endothelial diﬀerentiation | BM-MSCs | (19) |
| AT-MSCs | better diﬀerentiation capacity to sinus-like cells | BM-MSCs | (20) |
| Rat SM-MSCs | better myogenesis potential and higher expression of myoblast markers | Rat AT-MSCs and BM-MSCs | (21) |
| Human AT-MSCs | More myogenic differentiation | BM-MSCs | (22) |
| Fetal UC, BM, and AM-MSCs | More potential to produce functional cardiomyocytes | Adult AT-MSCs and BM-MSCs | (23) |

**References**

1. Vishnubalaji R, Al-Nbaheen M, Kadalmani B, Aldahmash A, Ramesh T. Comparative investigation of the differentiation capability of bone-marrow- and adipose-derived mesenchymal stem cells by qualitative and quantitative analysis. Cell Tissue Res. 2012 Feb 28;347(2):419–27.

2. Afizah H, Yang Z, Hui JHP, Ouyang H-W, Lee E-H. A Comparison Between the Chondrogenic Potential of Human Bone Marrow Stem Cells (BMSCs) and Adipose-Derived Stem Cells (ADSCs) Taken from the Same Donors. Tissue Eng. 2007 Apr;13(4):659–66.

3. Fu W-L, Zhang J-Y, Fu X, Duan X-N, Leung KKM, Jia Z-Q, et al. Comparative study of the biological characteristics of mesenchymal stem cells from bone marrow and peripheral blood of rats. Tissue Eng Part A. 2012 Sep;18(17–18):1793–803.

4. Rebelatto CK, Aguiar AM, Moretão MP, Senegaglia AC, Hansen P, Barchiki F, et al. Dissimilar Differentiation of Mesenchymal Stem Cells from Bone Marrow, Umbilical Cord Blood, and Adipose Tissue. Exp Biol Med. 2008 Jul;233(7):901–13.

5. Barlow S, Brooke G, Chatterjee K, Price G, Pelekanos R, Rossetti T, et al. Comparison of Human Placenta- and Bone Marrow–Derived Multipotent Mesenchymal Stem Cells. Stem Cells Dev. 2008 Dec;17(6):1095–108.

6. Montesinos JJ, Flores-Figueroa E, Castillo-Medina S, Flores-Guzmán P, Hernández-Estévez E, Fajardo-Orduña G, et al. Human mesenchymal stromal cells from adult and neonatal sources: comparative analysis of their morphology, immunophenotype, differentiation patterns and neural protein expression. Cytotherapy. 2009 Jan;11(2):163–76.

7. Bernardo ME, Emons JAM, Karperien M, Nauta AJ, Willemze R, Roelofs H, et al. Human Mesenchymal Stem Cells Derived from Bone Marrow Display a Better Chondrogenic Differentiation Compared with Other Sources. Connect Tissue Res. 2007 Jan 6;48(3):132–40.

8. Guillot P V., De Bari C, Dell’Accio F, Kurata H, Polak J, Fisk NM. Comparative osteogenic transcription profiling of various fetal and adult mesenchymal stem cell sources. Differentiation. 2008 Nov;76(9):946–57.

9. Taléns-Visconti R, Bonora A, Jover R, Mirabet V, Carbonell F, Castell J-V, et al. Hepatogenic differentiation of human mesenchymal stem cells from adipose tissue in comparison with bone marrow mesenchymal stem cells. World J Gastroenterol. 2006 Sep 28;12(36):5834–45.

10. ZHENG Y, GAO Z, XIE C, ZHU H, PENG L, CHEN J, et al. Characterization and hepatogenic differentiation of mesenchymal stem cells from human amniotic fluid and human bone marrow: A comparative study. Cell Biol Int. 2008 Nov;32(11):1439–48.

11. Lee H-J, Jung J, Cho KJ, Lee CK, Hwang S-G, Kim GJ. Comparison of in vitro hepatogenic differentiation potential between various placenta-derived stem cells and other adult stem cells as an alternative source of functional hepatocytes. Differentiation. 2012 Oct;84(3):223–31.

12. Zanini C, Bruno S, Mandili G, Baci D, Cerutti F, Cenacchi G, et al. Differentiation of Mesenchymal Stem Cells Derived from Pancreatic Islets and Bone Marrow into Islet-Like Cell Phenotype. Rameshwar P, editor. PLoS One. 2011 Dec 16;6(12):e28175.

13. Marappagounder D, Somasundaram I, Dorairaj S, Sankaran R. Differentiation of mesenchymal stem cells derived from human bone marrow and subcutaneous adipose tissue into pancreatic islet-like clusters in vitro. Cell Mol Biol Lett. 2013 Jan 1;18(1):75–88.

14. Wu L-F, Wang N-N, Liu Y-S, Wei X. Differentiation of Wharton’s jelly primitive stromal cells into insulin-producing cells in comparison with bone marrow mesenchymal stem cells. Tissue Eng Part A. 2009 Oct;15(10):2865–73.

15. Yan Z-J, Hu Y-Q, Zhang H-T, Zhang P, Xiao Z-Y, Sun X-L, et al. Comparison of the Neural Differentiation Potential of Human Mesenchymal Stem Cells from Amniotic Fluid and Adult Bone Marrow. Cell Mol Neurobiol. 2013 May 12;33(4):465–75.

16. Balasubramanian S, Thej C, Venugopal P, Priya N, Zakaria Z, SundarRaj S, et al. Higher propensity of Wharton’s jelly derived mesenchymal stromal cells towards neuronal lineage in comparison to those derived from adipose and bone marrow. Cell Biol Int. 2013 May 1;37(5):507–15.

17. Datta I, Mishra S, Mohanty L, Pulikkot S, Joshi PG. Neuronal plasticity of human Wharton’s jelly mesenchymal stromal cells to the dopaminergic cell type compared with human bone marrow mesenchymal stromal cells. Cytotherapy. 2011 Sep;13(8):918–32.

18. Zhang H-T, Liu Z-L, Yao X-Q, Yang Z-J, Xu R-X. Neural differentiation ability of mesenchymal stromal cells from bone marrow and adipose tissue: a comparative study. Cytotherapy. 2012 Sep;14(10):1203–14.

19. Chen M-Y, Lie P-C, Li Z-L, Wei X. Endothelial differentiation of Wharton’s jelly–derived mesenchymal stem cells in comparison with bone marrow–derived mesenchymal stem cells. Exp Hematol. 2009 May;37(5):629–40.

20. Huang C, Song T, Wu P, Chen Y, Fan X, Chen H, et al. Differentiation potential of human mesenchymal stem cells derived from adipose tissue and bone marrow to sinus node-like cells. Mol Med Rep. 2011 Oct 3;5(1):108–13.

21. Meligy FY, Shigemura K, Behnsawy HM, Fujisawa M, Kawabata M, Shirakawa T. The efficiency of in vitro isolation and myogenic differentiation of MSCs derived from adipose connective tissue, bone marrow, and skeletal muscle tissue. Vitr Cell Dev Biol - Anim. 2012 Apr 7;48(4):203–15.

22. STERN-STRAETER J, BONATERRA GA, JURITZ S, BIRK R, GOESSLER UR, BIEBACK K, et al. Evaluation of the effects of different culture media on the myogenic differentiation potential of adipose tissue- or bone marrow-derived human mesenchymal stem cells. Int J Mol Med. 2014 Jan;33(1):160–70.

23. Ramkisoensing AA, Pijnappels DA, Askar SFA, Passier R, Swildens J, Goumans MJ, et al. Human Embryonic and Fetal Mesenchymal Stem Cells Differentiate toward Three Different Cardiac Lineages in Contrast to Their Adult Counterparts. Covas DT, editor. PLoS One. 2011 Sep 9;6(9):e24164.
